# GASICA: generic automated stress induction and control application design of an application for controlling the stress state

**DOI:** 10.3389/fnins.2014.00400

**Published:** 2014-12-08

**Authors:** Benny van der Vijgh, Robbert J. Beun, Maarten van Rood, Peter Werkhoven

**Affiliations:** ^1^Buys Ballot Laboratory, Department of Information and Computing Sciences, Utrecht UniversityUtrecht, Netherlands; ^2^Department of Neurology and Neurosurgery, University Medical Center UtrechtUtrecht, Netherlands

**Keywords:** GASICA, stress state, stress state control, psychological stressor, physiological response, feedback model, stressor game

## Abstract

In a multitude of research and therapy paradigms it is relevant to know, and desirably to control, the stress state of a patient or participant. Examples include research paradigms in which the stress state is the dependent or independent variable, or therapy paradigms where this state indicates the boundaries of the therapy. To our knowledge, no application currently exists that focuses specifically on the automated control of the stress state while at the same time being generic enough to be used in various therapy and research purposes. Therefore, we introduce GASICA, an application aimed at the automated control of the stress state in a multitude of therapy and research paradigms. The application consists of three components: a digital stressor game, a set of measurement devices, and a feedback model. These three components form a closed loop (called a *biocybernetic loop* by Pope et al. (1995) and Fairclough (2009) that continuously presents an acute psychological stressor, measures several physiological responses to this stressor, and adjusts the stressor intensity based on these measurements by means of the feedback model, hereby aiming to control the stress state. In this manner GASICA presents multidimensional and ecological valid stressors, whilst continuously in control of the form and intensity of the presented stressors, aiming at the automated control of the stress state. Furthermore, the application is designed as a modular open-source application to easily implement different therapy and research tasks using a high-level programming interface and configuration file, and allows for the addition of (existing) measurement equipment, making it usable for various paradigms.

## Introduction

For various cognitive and affective (neuroscience) research and therapy purposes, information on the internal stress state of patients and participants and, desirably, exerting control over this stress state, is important. For instance, during exposure therapy it is of importance to have information concerning the internal stress state of a patient, and, based on the inclination of the therapist, either control this stress state to keep psychological responses to the exposed stimuli within certain boundaries, or leave the state as is, and use the received information to determine which states are optimal for a specific therapy. The same holds for various cognitive and affective research, e.g., memory and risk-taking research, where the stress state of a participant can act as a confounding factor during the research.

Currently, to our knowledge, no application exists that focuses specifically on the automated control of the stress state while at the same time being generic enough to be used in various therapy and research purposes. There exist tasks that allow to adjust the presented stressor intensity, for example the Montreal Imaging Stress Task (MIST, Dedovic et al., 2005). However, this adjustment cannot be done real-time: the intensity can only be set at the start of the task, hereby not allowing control over the stress state during the therapy or research paradigm. Therefore, we propose a new application in this paper, dubbed GASICA: *Generic Automated Stress Induction and Control Application*. This application is aimed at automated control of the internal stress state in various therapy and research paradigms by online and continuous monitoring of the stress state.

The stress state generally refers to the current state of stress present in the subject (i.e., a patient or participant). However, over the years, stress research has produced a myriad of definitions for the concept of stress, expressing a wide variety of views on the matter. This variety has emerged due to several reasons, such as the multidisciplinary nature of stress research and the developing view on stress in the past decades.

Our definition of stress is based on the definition by Newport and Nemeroff (2002) where stress is defined as any challenge to homeostasis of an individual that requires an adaptive response of that individual. In order to use this definition in the construct of GASICA, we use it in the following form: “*Stress is the state resulting from the ensemble of responses that are aimed at (facilitating) restoration and/or maintenance of (psychological) homeostasis to internal or external stimuli that present (perceived) challenges to this (psychological) homeostasis.*”

To be more specific, the stimulus presenting the challenge to (psychological) homeostasis will be referred to as the *stressor*, and the response to this challenge referred to as the *stress response*. In par with the interactional approach on stress (Jones and Bright, 2001), we furthermore identify variables influencing the relation between the stressor and stress response, referred to as *intervening variables*.

Stressors, stress responses and intervening variables can be divided into different categories. With regard to stressors, two of the categorizations that are often made and that are relevant to our application, are the division of stressors in *physical* and *psychological* stressors, and in *acute* and *chronic* stressors (see, amongst others, Jones and Bright, 2001; Dickerson and Kemeny, 2004). Physical stressors are in general defined as metabolically demanding stressors, such as physical exercise or a cold pressor task, where the hand of a subject is cooled down. Psychological stressors are in most cases defined as non-metabolically demanding stressors, for example as used in the Trier Social Stress Test, in which a subject is asked to perform a public speech task and mental arithmetic (Kirschbaum et al., 1993). The division between acute and chronic stressors refers to the length a stressor is presented. The precise boundary between these categories varies in the literature, although a consensus can be discerned on the stressor lengths on the ends of the spectrum: stressors that are presented within the realm of minutes are regarded as acute stressors and stressors that are present for weeks or longer will be regarded as chronic.

The stress response can also be divided into different categories. An often-made categorization is the distinction between *physiological*, *psychological* and *behavioral* stress responses (see, amongst others, Jones and Bright, 2001; Lovallo, 2005). Physiological stress responses can include, among others, alterations in heart rate (variability), cerebral activity, and electrodermal activity, while psychological responses typically include alterations in affect and cognition, and behavioral responses include alterations of the exhibited behavior.

Intervening variables have been divided into a broad array of different categories in the literature, depending on the aim of the categorization made. However, there are several categories that are prevalent throughout this literature. These categorizations include, amongst others, *individual difference variables*, including variables such as age, gender and personality type, and *environmental variables*, including variables such as the surrounding temperature.

Within the stressor and stress response categories we introduce a further distinction between *type* and *form*. This distinction will aid us in the further design and description of GASICA. We define within this context stressor types to refer to the manifestation of a stressor, i.e., the concrete entity that produces the stressor: for example a public speaking task, a mathematical task, or a digital game producing a stressor. Stressor form will refer to the kind of stressor(s) that is or are presented through this stressor type, for example workload, social-evaluative threat (the possibility of being negatively judged by others), or frustration.

Analogous we introduce the term stress response type to refer to the manifestation of physiological stress response(s), such as heart rate responses (e.g., an elevated heart rate), or cortisol responses (e.g., an increased cortisol level in the blood). Stress response form refers to the kind of physiological response system the response type is originating from, e.g., stress responses of the form *sympathetic* are originating from the sympathetic nervous system (responsible for the electrodermal stress response type), and *hemodynamic* responses are responses of the hemodynamic response system (responsible for changes in blood pressure).

The difference between the concepts stress and *arousal* remains an interesting point of discussion in the breadth of the stress research community. To our knowledge there is currently no consensus on how to separate the two. Of the different views that are currently present on this matter, we concur with the view expressed by Day and Walker (2007), in that the difference between these two concepts needs to be sought rather in a top-down characterization of qualitative appraisal, opposed to a bottom-up characterization of physiological responses. Based on this thought Day and Walker propose three ways in which stress differs from arousal. The first way is that stress differs from arousal in terms of the qualitative appraisal that precedes it. The second is that stress is only elicited by aversive challenges. And the third is that there is a difference in the resulting physiological state between stress and arousal, which the authors state based on, amongst others, memory research. However, the authors indicate that the exact differences between these physiological states are currently not elucidated.

In GASICA we will utilize an *acute psychological* stressor (i.e., a stressor that falls both in the psychological stressor category and the acute stressor category) and we will measure *physiological* stress responses (i.e., stress responses in physiological signals). An *acute* stressor is used since, for practical reasons, a stressor cannot be presented for days or weeks within the application, as this will not be suitable for most therapy and research paradigms. Furthermore, a *psychological* stressor is used, as this allows for more flexibility than a physical stressor. This because the adjustment of physical entities, e.g., changing the temperature of water, is more cumbersome and less flexible than the adjustment of a psychological stressor, allowing a more generic application. A *physiological* stress response is used as an indication of the internal stress state as this category of stress response is the best objectively measurable and quantifiable of the three different stress response categories. Therefore, in the context of our application, we further refine our definition of the stress state, defining it as the current overall stress response in spontaneously generated (neuro)physiological signals, i.e., the current sum of different (neuro)physiological stress response types.

**Figure 1:**
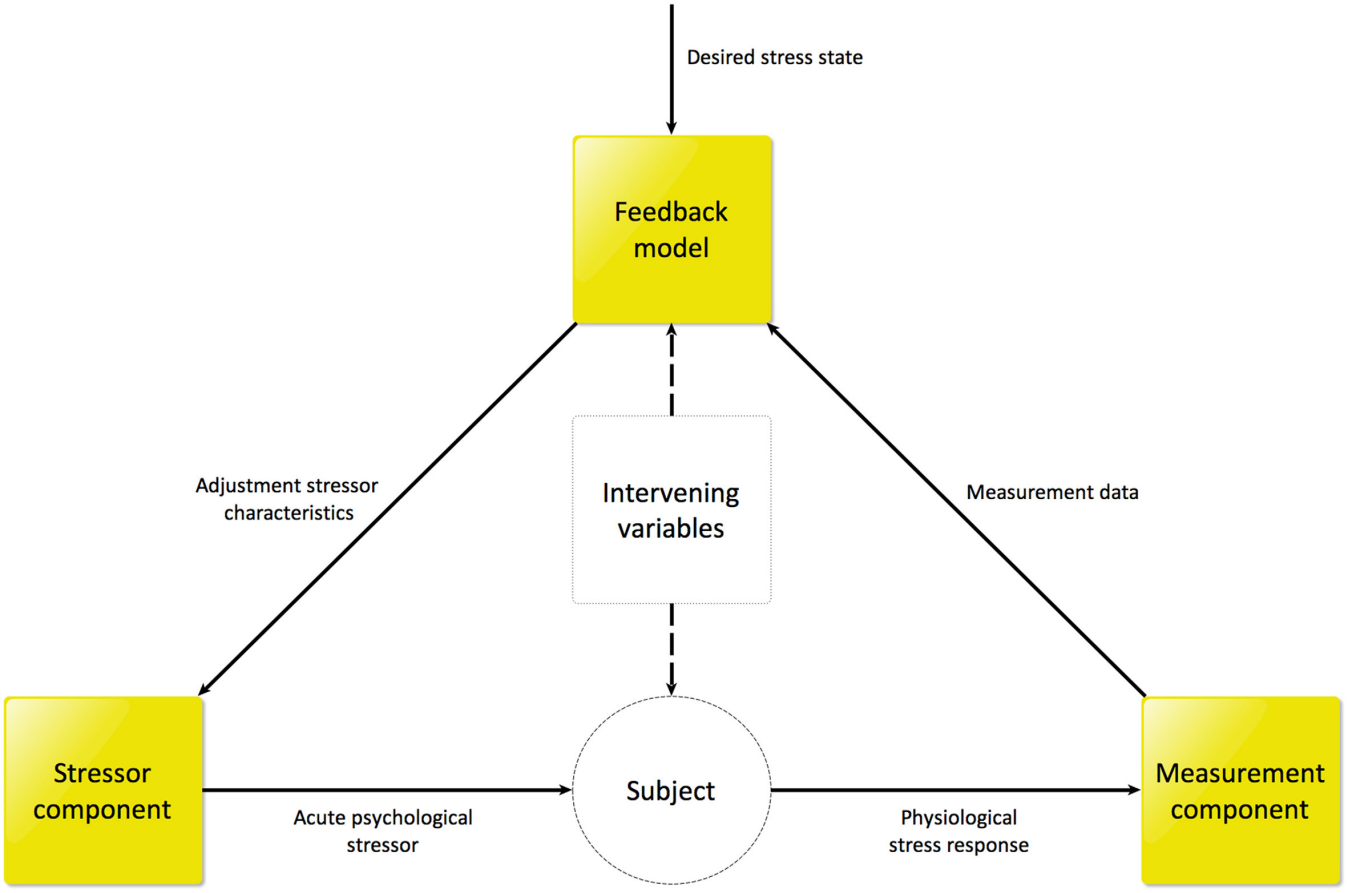
**Schematic overview of GASICA**.

In order to achieve the aim of automated controlling of the stress state, it is essential to control the stressor intensity *causing* the current stress state. Therefore, our proposed application consists of three components, depicted in Figure 1:

1. A stressor, allowing to induce stress responses.
2. Set of measurement devices, allowing for continuous and online measurement of several (neuro)physiological signals.
3. Feedback model that derives the current internal stress state of the human subject based on the measured (neuro)physiological signals and adjusts the stressor characteristics based on this derived state and the desired stress state in the current paradigm.

In this paper, we will describe the design of GASICA by discussing each of the three separate components in detail in the upcoming sections.

## GASICA

In this section we will further design the different components in our application (i.e., the stressor component, measurement component and the feedback model) using a top-down approach. In this design process we aim to maximize the diagnosticity, sensitivity, and reliability of GASICA, i.e., we aim to maximize the extent to which we are able to measure different levels psychological stress across inter- and intra-individual differences (for a detailed treatment of these concepts, see Fairclough, 2009). As the category of the utilized stressor and stress response are already determined, we will first determine the form and type of the utilized stressor and stress responses, using requirements formulated based on our aim to continuously measure and exert control over the stress state in various research and therapy paradigms. Subsequently we will determine the characteristics of the different components using a recent meta-analysis we conducted (van der Vijgh et al., 2015) as guideline. Finally we will determine the instantiations and/or implementations of the components, using sets of requirements and the meta-analysis as guidelines. This process is described in the following sections for the different components separately.

### Component 1: stressor

#### Stressor type

For the first component, the stressor, we utilize an acute psychological stressor. To determine the *type* of acute psychological stressor best suited for use in our application we formulated four requirements:

1. Multidimensionality
  The stressor should allow for presentation of multiple stressor forms (e.g., workload, emotion induction, and frustration) to make it suitable for multiple therapy and research designs.
2. Adjustability
  In order to allow for presenting different stressor intensities, the stressor characteristics should be adjustable, in a way that results in different stressor intensities. Furthermore, through this adjustment it should also be possible to adjust the stressor form presented.
3. Real-time
  The adjustment of the stressor characteristics should be possible in real-time, in order to respond to changing stress states. In this context, real-time refers to the realm of seconds, so that the application can adjust the stressor within a couple of seconds when needed.
4. Continuity
  To allow continuous control over the stressor that is presented, it should be possible to both present and adjust the stressor continuously for a prolonged period of time.

When we look at acute psychological stressors to match against these requirements, we find a plethora of different stressor types. In an influential meta-analysis by Dickerson and Kemeny acute psychological stressors were subdivided in four mutually exclusive types: public speaking and verbal interaction tasks, cognitive tasks, emotion induction procedures, and noise exposure tasks (Dickerson and Kemeny, 2004).

Public speaking and verbal interaction tasks refer to tasks in which subjects have to verbally interact with other human subjects, such as in interviews or through public speaking. The stressor intensities of these tasks are hard to adjust, especially in real-time, and the tasks present a mostly one-dimensional stressor form, being mostly social stress (social-evaluative threat).

Cognitive tasks are tasks such as arithmetic tasks, the Stroop task, vigilance-reaction time tasks, and analytical tasks, e.g., puzzles. These kind of tasks are, especially when presented digitally, well adjustable, also in real-time, and can be presented continuously. However, these tasks also present a mostly one-dimensional stressor form, being workload.

Emotion inducement tasks are tasks that present emotion-eliciting material that elicit a negative affective state, such as the viewing of aversive pictures or film. These tasks are by definition one-dimensional in the sense that these only induce emotion as stressor form.

Noise exposure tasks exist of the presentation of loud noises. These kinds of tasks are also by nature one-dimensional.

Another widely used type of acute psychological stressor not discussed in this analysis consists of a combination of the latter three stressor types, hereby alleviating the limitation of one-dimensionality pertaining to the individual stressor types: a *digital stressor game*, i.e., a digital game producing a stressor. In a recent meta-analysis 5448 articles were found when using search phrases to find studies utilizing this type of stressor^1^, indicating the widespread use of this type of stressor (van der Vijgh et al., 2015). We adhere to the definition of a game used in this analysis: a game is defined as a type of play activity conducted in the context of a pretended reality, in which the player(s) try to achieve at least one arbitrary, nontrivial goal by acting in accordance with rules (Adams, 2010). Key elements in games are players, (inter)action, environment, goals, and rules. In a game, players interact with entities in the environment or with other players in accordance with a set of rules in order to achieve a set goal. In the case a player controls a specific entity, this entity is referred to as the avatar. Game characteristics are characteristics of any (part of) of the key elements or the game as a whole, such as the game type, the presence of game music or time pressure, or the amount of aversive stimuli present in the digital game.

As in digital games many, or even all, of the key elements are taken over by computer technology, this provides possibilities that allow to *adjust* the stressor intensity, in *real-time*, by adjusting the game characteristics of the digital game stressor. Also, by adjusting these game characteristics, it is possible to present *multi-dimensional* stressor forms, *continuously*, satisfying our requirements. Even more, a (digital) game provides a *narrative*, the pretended reality in which the goal that is set is tried to be achieved. This narrative provides possibilities to conceal the adjustments made to the stressor characteristics and changes between stressor forms from the subject by presenting these adjustments as part of the narrative. The presence of a narrative also provides a way to incorporate different research and therapy paradigms in the application, as it allows to create a specific narrative for each specific paradigm. Given these properties (adjustable, real-time, continuity, multidimensional, and a narrative), a digital game stressor is selected as the stressor type in our application.

#### Characteristics

In order to present different stressor intensities using this type of stressor, it is essential to have insight in which digital game characteristics elicit physiological stress responses, i.e., alter the stress state, and to what degree. Additionally, the effects of adjustments of these characteristics on the stress state should be predictable.

In the same meta-analysis by van der Vijgh et al. (2015), the relation between digital game characteristics and physiological stress responses is analyzed. Using meta-regression, this analysis identified four stressor game characteristics, presenting different stressor forms, that significantly moderated the physiological stress responses in a predictable and consistent manner. This indicates that these characteristics can be used to elicit stress responses and that adjustments of these characteristics are expected to result in predictable changes of the stress state. These four characteristics will therefore be instantiated in the digital game:

1. Aversive stimuli
  The first game characteristic identified is the presence and intensity of *aversive stimuli* in the digital game, such as the visual or auditory presentation of scenes of violence, blood, or gore. Aversive stimuli have been found to elicit physiological stress responses both inside as well as outside a digital game context. Outside the context of a game, the presentation of aversive stimuli such as in a picture rating task (Stegeren et al., 2008), passive viewing of aversive pictures (Sokhadze, 2007) or film viewing containing aversive stimuli (Miller et al., 1995) has been found to induce physiological stress responses such as alterations in heart rate, electrodermal activity and frontal EEG activity. Within the context of a game aversive stimuli can, for example, include the presence of violence, blood and gore (Hebert et al., 2005), and torture (Tafalla, 2007), also inducing physiological stress responses (Carnagey et al., 2007). This characteristic presents a stressor of the form emotion induction, as it induces a negative affect.
2. Realism
  This characteristic concerns the amount of *realism* presented in the digital game. This refers to the degree to which a subject will identify the presented stimuli as realistic. This characteristic does not present a separate stressor form in itself, but rather heightens the immersion, resulting in heightened physiological stress responses. Several studies have found the amount of realism in digital games to be related to resulting physiological stress responses. For example, Ivory and Kalyanaraman (2007) found that more technologically advanced, although otherwise comparable, digital games elicited higher electrodermal stress responses and Barlett and Rodeheffer (2009) showed that more realistic digital games significantly heighten the heart rate stress response.
3. Game music
  *Game music* refers to whether or not music is presented in the game. A recent overview provided by Sokhadze (2007) makes clear that although there are inconsistent results to be found, music has the potential to elicit physiological stress responses. Examples include work by Nyklicek et al. (1997), who found significant differences in both cardiovascular and respiratory variables in response to different fragments of music and white noise. Other physiological stress responses, such as in skin temperature, have also been found by, amongst others, McFarland (1985), who found that music with different valence and arousal (subjectively determined) have different effects on skin temperature. Game music induces emotion, the same stressor form as aversive stimuli.
4. Game type
  This characteristic concerns the *game type*, the type of digital game. Examples of different game types include action, adventure, strategy and management, role playing games (RPG), simulation or board and card games (Ritterfeld et al., 2009). In the meta-analysis, it was found that puzzle games induce the highest physiological stress responses. Game type in itself does not present a stressor form, as it acts as a container characteristic: the characteristics that are contained within a specific game type are responsible for the resulting stressor form.

Besides these four characteristics, several other game characteristics were identified in this meta-analysis to be related to physiological stress responses, but were not used in the meta-regression because these could not be objectively qualified or were not reported sufficiently in the included studies. As we aim to design an application that can be applied in various therapy and research paradigms, we aim to include as many different game characteristics as possible, allowing a wider range of stressor forms to be presented and thereby providing greater flexibility for use in more paradigms. Therefore, we screened these characteristics that were excluded from the meta-regression to see if these were fit to use in our stressor. Of these additional characteristics, three digital game characteristics are selected to be incorporated in the digital game, based on the property of these three characteristics to be adjustable during execution of the application, bringing the total of game characteristics used in our digital game stressor to seven:

(5) Time pressure
  *Time pressure* refers to the presence of limited time before a certain goal has to be achieved. Studies utilizing time pressure paradigms (Wahlstrom et al., 2002) have consistently been found to elicit physiological stress responses. The stressor form time pressure presents is workload, as it increases the demand placed on the subject.
(6) Sound level
  *Sound level* concerns the sound level at which auditory stimuli are presented during the game. This characteristic presents a stressor of the form noise induction and it has been shown that high sound levels, mostly studied at 75 dB and above, elicit physiological stress responses (Smith et al., 1997; Selander et al., 2009).
(7) Disabling of input
  *Disabling of input* refers to the disabling of the control the subject has in the game making it harder to achieve the goals set, resulting in frustration and physiological stress responses (Reuderink et al., 2009).

In Table 1 an overview is given of the seven included stressor game characteristics and the stressor forms these present.

**Table 1:** **Overview of stressor game characteristics, with presented stressor form, instantiation within the stressor game, the way the instantiations fit in the narrative and how these are adjusted in order to control the stress state**.

**Stressor game characteristic**	**Stressor form**	**Instantiation of characteristic**	**Narrative**	**Adjustment**
Aversive stimuli	Emotion induction	IAPS and IADS	Malfunctioning of suit: unwanted presentation of pictures and sounds.	Absence/Presence of pictures and sounds, and selection of pictures and sounds of different values of new scale.
Realism	Not applicable	Point of view	Malfunctioning of suit: point of view is adjusted.	Point of view is either first or third person.
Game music	Emotion induction	Music samples	Malfunctioning of suit: unwanted presentation of music.	Absence/Presence of music, and selection of samples of different values of new scale.
Game type	Not applicable	Not applicable	Not applicable	Not applicable
Time pressure	Workload	Countdown	Uncle needs next notes quickly.	Absence/Presence of countdown and starting number.
Sound level	Noise induction	Sound level auditory stimuli	Malfunctioning of suit: volume built-in radio is adjusted.	Different sound levels.
Disabling input	Frustration	Disabling input	Malfunctioning of suit: movement is restricted.	Absence/Presence of disabling of input: if disabled, which key is disabled.

#### Game design

The stressor game is designed around the seven selected game characteristics, aiming to provide a narrative through which the game characteristics can be adjusted as part of the narrative, reducing the possibility of subjects noticing the adjustments as being part of the stress state control method.

The game is designed as a 3D puzzle game because the same meta-analysis (van der Vijgh et al., 2015) indicated that this game type elicited the highest stress response of the analyzed game types. The narrative provided is that an adolescent boy or girl (i.e., the avatar, the entity that is controlled by the subject, gender is not made explicit) has to find notes that are scattered across the house and garden of his or her uncle, an inventor and scientist, who needs these notes within a given amount of time in order to finish his work. The boy/girl sets out to find these notes wearing one of the latest inventions by this uncle, a heavy suit that should help the subject to find these notes, outfitted with a radio and head up display that can present auditory and visual stimuli to the wearer. However, this suit turns out not to work as expected, malfunctioning from time to time, resulting in, for example, the possible presentation of images or sounds, and the restriction of movement. This suit allows the game characteristics to be altered as needed without the subject registering this as a conscious adaptation of the game, as these alterations are presented as malfunctions of the suit the subject is wearing. Furthermore, the narrative serves to heighten immersion and reduce boredom and annoyance by motivating the subject to keep participating in the current paradigm. The stressor game is designed to exclude fast-paced motor action or complicated cognitive tasks in order to prevent uncontrolled elements of the stressor to induce physiological reactions. Furthermore, the setting of the game does not include political or ideological content, to prevent unwanted side-effects, nor fast-paced visual or auditory sequences (e.g., no flashing lights) to prevent uncontrolled stress responses and to make sure the digital game can be utilized in multiple paradigms.

**Figure 2:**
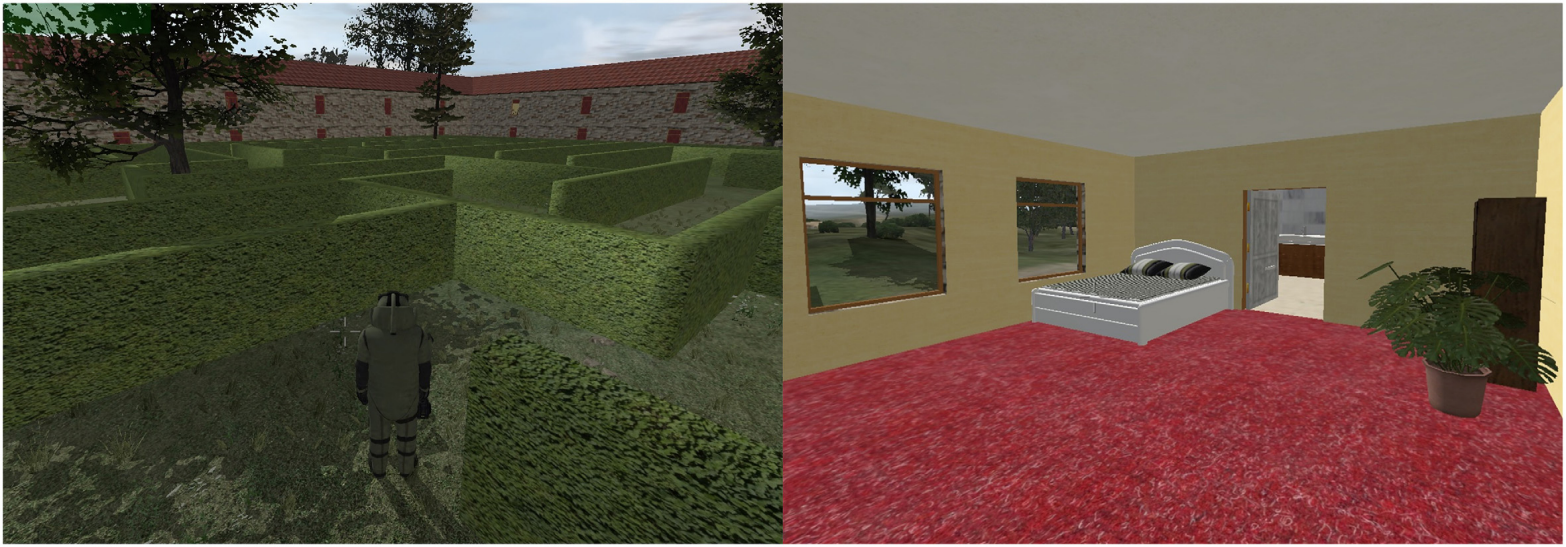
**Impression of condition 1 and 2 in the stressor game, respectively**.

Within the game there are two separate conditions: a *fitting* and *manipulation* condition. The fitting condition serves to fit an individual feedback model for each subject. This condition consists of a maze (the garden of the uncle) that presents a homogeneous environment that is suited for the fitting of the feedback model (for more details, see Section Fitting). To prevent unwanted stressor effects of getting lost in the maze we placed trees as landmarks to help subjects find their way. The manipulation condition serves to utilize the fitted feedback model to control the stress state and to present tasks or therapy elements (for more details, see Section Overview). This condition is placed inside the house of the uncle. Impressions of both conditions are given in Figure 2.

#### Instantiations of characteristics

Within this design, the seven selected stressor game characteristics are instantiated in order to control the stress state. We chose instantiations that allowed changes to the characteristics to be immediately noticeable. An overview of the instantiations and how these fit in the narrative of the stressor game is given in Table 1.

Aversive stimuli are presented using the International Affective Picture System (IAPS) (Lang et al., 1988) and the International Affective Digitize Sounds (IADS) (Bradley and Lang, 1999), standard sets containing over 1000 pictures and 167 sounds respectively. These sets are scored on valence, arousal and dominance by subjects in an on-going sequence of studies, currently containing the scoring from over 18 separate studies. By summing the scores on inversed dominance, valence, and arousal, we derived a new scale in which a low value corresponded with images and sounds scored as unhappy, arousing and being controlled and higher values corresponded with images scored as happy, non-arousing and being in control. Pictures are presented full screen to achieve maximal effect, the subject is explained that this is displayed on the display incorporated in the suit, and sounds are presented over the built-in radio, both are malfunctions of the suit.

Realism is altered through changing the point of view to either 1st or 3rd person view, i.e., the view the subject has in the stressor game is either through the eyes of the avatar or from above, just beyond the avatar, having view of the complete avatar. Altering this view has been found by Dahlquist et al. (2010) to alter presence: in 1st person view a greater sense of presence was reported. This adjustment is also presented as a malfunction of the suit.

As game music has been found to induce different responses, it is hard to determine how to instantiate this characteristic. Based on the review provided by Sokhadze (2007) we used the results from the study by Nyklicek et al. (1997) as a basis, as this study was found to be the most comprehensive study, and having clear significant results. These results indicate the possibility to elicit different emotional states (recognized based on physiological responses), utilizing music excerpts that are characterized by valence and arousal dimensions. Based on these findings we chose to use a standard set of music excerpts, scored on several dimensions, including valence, and arousal, by 116 subjects (Eerola and Vuoskoski, 2011). We derived a new scale by summing the values for valence, tension, and energy. In this scale low values correspond to negatively valenced, tensed, and energetic music, and high values correspond to positively valenced, relaxed, and calm music. Music is presented over the built-in radio, in the narrative of the game this is explained as a malfunction of the suit.

The game type will not be altered during the execution of the game, and is set as a puzzle game. This is because changes in the game type has also consequences for the narrative and game design, which is undesirable.

Time pressure will be applied through a countdown presented to the subject indicating that he or she needs to find the next note before the timer reaches zero. This timer can be turned on or off and can start at any given number of seconds. Within the narrative of the game the time pressure is applied through the uncle announcing over the radio that he needs new notes quickly.

Sound level is adjusted by presenting the auditory stimuli in different intensities, this is presented as a malfunction of the radio in the suit.

Disabling input is induced by (partially) disabling control by disabling the keys needed to control the game. In the narrative of the game this is also a malfunction of the suit the subject is wearing.

Each of the stressor characteristics instantiations can be presented either *transient* or *state-wise*, as can be determined by the experimenter or therapist. This refers to the duration an adjustment of the instantiation is present: either for a set duration (that can be determined by the experimenter or therapist) or constantly, until it is adjusted again.

An additional strength of the included stressor game characteristics and the respective instantiations is that these present stressor forms have a high degree of ecological validity. This is due to fact that these stressors have a high correlation with stressors found in real life. Exemplars are the real-life depictions of aversive visual and auditory stimuli of the IAPS and IADS that are used, and the presentation of time pressure on task-completion, as presented through the countdown in the stressor game.

#### Implementation

In order to implement the stressor game we aimed to utilize a software environment which fulfilled three requirements:

1. Data exchange
  It must be possible to both import and export information into and from the environment during execution. Import is needed to receive information from the feedback model, in order to receive which game characteristics will be adjusted in what way. Export is needed to send markers to the measurement component or any additional hardware.
2. Characteristic implementation
  All of the selected game characteristics instantiations must be implementable in the environment. For example, the environment must allow for a high level of realism in order to be able to vary the amount of realism presented.
3. Real-time adjustment
  The implemented game characteristic instantiations must be real-time adjustable.
4. High-level programming interface
  In order to make the application suitable for different therapy and research paradigms, it is needed to have a high-level programming interface that allows to easily implement the needed research or therapy tasks within the application.

Based on these requirements the software environment Virtual BattleSpace 2 (VBS2) by Bohemia Interactive was selected (Simulations, 2011). This is a 3D simulation environment that allows for complete control over the simulation to adjust the relevant game characteristics and fulfill the above requirements. The subjects control the stressor game with the four directional keys of a standard alphanumeric keyboard.

### Component 2: measurement

#### Stress response types

In the measurement component we include a multitude of physiological stress response types, both to provide information suited for a variety of research and therapy paradigms as well as to gain as much information as possible regarding the current stress state. In order to determine which types and corresponding measurements are suitable for inclusion we drafted six requirements, the first three concerning the stress response types, the latter three concerning the corresponding measurements, given below:

1. Responsitivity
  The stress response type should respond to the selected stressor game characteristics.
2. Response consistency
  In order to succeed in achieving the intended effect in the measured stress response upon adaptations of the stressor, the response must be consistent within a subject to a (digital game) stressor (adaptation), both quantitatively and temporally. This entails the response should have a consistent sign (either increasing or decreasing) between repeated presentations of an identical stressor game characteristic, and this response must occur within the same time span between repeated presentations. This consistency is only required for identical circumstances. For example the response is not required to be consistent between cases where in one of the cases the stress response type is already at a physiological possible maximum or minimum.
3. Low response latency
  In order to be able to measure the effect of stressor characteristic adjustment and allow for any subsequent adjustments, the stress response must emerge after such an adjustment with the least amount of delay as possible. In practice, this requires the latency to be within the realm of seconds, in order to reliably determine the effect of a presented stressor.
4. Measurement continuity
  The measurement must be applicable continuously in order to keep continuous track of the stress state and the effects of stressor adjustments.
5. Measurement inertion
  In order to reliably relate the stress responses to adjustments in the stressor game, the measurement must be applicable without inducing an additional stress response or disturbing the experiment or therapy.
6. Measurement fMRI compatibility
  In order to allow usage of the application in additional research and therapy paradigms, the measurement must be applicable inside a MRI scanner, without disturbing (f)MRI imaging, allowing the use of the application in combination with this technique.

We compiled a list of all stress response types and corresponding measurements encountered in the meta-analysis on digital game characteristics utilized in the previous section (van der Vijgh et al., 2015), as this provides an exhaustive overview of the kind of measurements performed with stressor games in the past 36 years. We reviewed these types and measurements to match these against the above requirements. The results hereof are given in Table 2, using the same numbering of the requirements as above. Table 2 contains 13 stress response types and corresponding measurements that meet all requirements. Although we aim for a multitude of measurements, including all these stress response types will require six different measurement devices^2^, which presents practical problems and is not expected to provide additional information concerning the overlap in the different stress response types and forms. Therefore, to reduce the number of needed measurement devices, we look at the stress response *form* the different stress response types have.

**Table 2:**
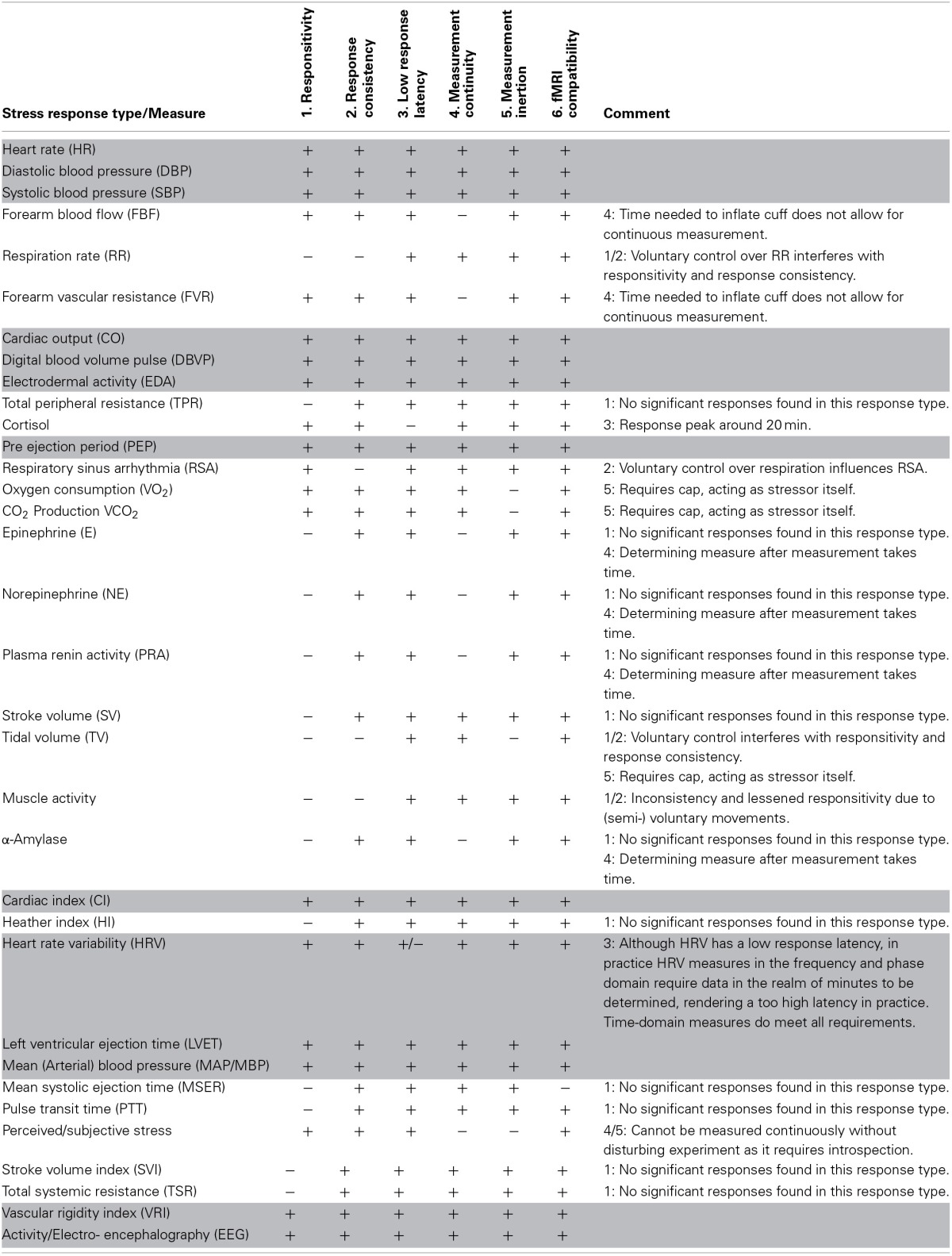
**Overview of stress response types and corresponding measurements matched against requirements for determining the utilized stress response types in the measurement component**.

A plus sign (+) indicates that the requirement of the respective column is met, a minus sign (−) indicates this requirement is not met. In the latter case, the comment column indicates why this is the case. Rows corresponding to stress response types and measurements that meet all requirements are given in gray.

The types are either of the cardiac response form (measured using ECG and ICG), hemodynamic response form (measured with blood pressure monitor and photophlesmograph), stress responses stemming from sympathetic activation (measured using electrodes), and the neural stress form, measured using electroencephalography (EEG). In order to reduce the number of needed measurement devices, we chose one measurement from each stressor form by selecting the measurement of the stress response types with the highest mean effect. These mean effect sizes^3^ are taken from the meta-analysis in which these stress response types were analyzed (van der Vijgh et al., 2015). For the different stress response forms and measurement combinations, these effect sizes are given in Table 3. Given this approach, we select to measure heart rate and heart rate variability (using ECG), systolic and diastolic blood pressure (using a blood pressure monitor), the electrodermal stress response type (electrodes) and the neural response using EEG.

**Table 3:**
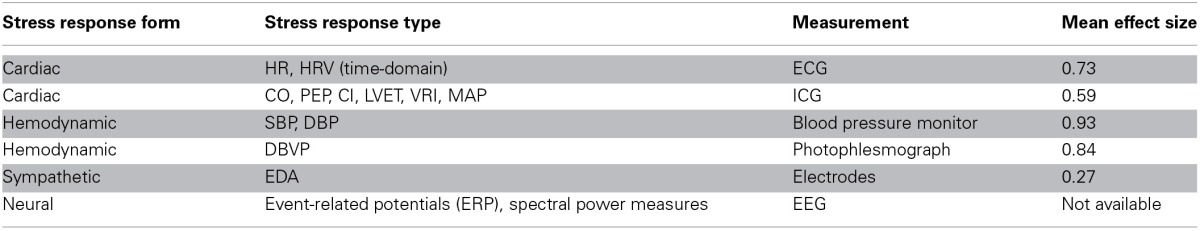
**Overview of stress response types fulfilling all requirements**.

Gray rows indicate selected types and forms for use in application.

#### Implementation

In order to implement the measurement component we aimed to utilize hardware and software that fulfilled three requirements:

1. fMRI compatible
  One of the requirements on the stress response types is that is possible to perform the measurement within a fMRI environment. Therefore, this requirement is extended to the hardware and software of the implementation as well.
2. Continuous
  We selected the stress response types amongst other things on the possibility to measure these continuously, therefore the hardware used to perform the measurement of these types also needs to be able to measure continuously.
3. Broad spectrum
  In order to make the application suitable for multiple research and therapy paradigms we select hardware and software that is easily extendable with measurement equipment for additional stress response types or the measurement of other dependent physiological variables, such as the use of EEG.

We were not able to find a manufacturer that provided interconnected equipment that fulfilled all our requirements and also measured all selected stress response types at the same time. We selected the system that fulfilled all three requirements and was able to measure the most stress response types at the same time. This is the fMRI-compatible equipment by Biopac Systems Inc. that allows continuous measurement of all our selected stressor types, except EEG. This results in the use of heart rate (variability), blood pressure and electrodermal activity measurements in the measurement component. The base station of this equipment, the *MP150*, allows easy extension with additional equipment through a plug-and-play interface which allows the plugging in of additional equipment by the same manufacturer, that is automatically recognized by the accompanying software, *Acqknowledge*. This ensures easy extension of the utilized stressor types, making the application suited for additional paradigms.

For the measurement of heart rate and variability with ECG we use the ECG 100C MRI amplifier with disposable electrodes in a lead-II type ECG. For the measurement of the electrodermal response we use two disposable electrodes on the hand or foot connected to an EDA 100C MRI amplifier. For measuring systolic and diastolic blood pressure we use a small pressure pad on the thumb or elbow that detects these measures using continuous pressure, allowing for continuous measurement, connected to an HLT-100C amplifier. This method utilizes an additional software package for extraction of the systolic and diastolic blood pressure from the raw blood pressure signal, *Caretaker*, which transfers the measurement data to the *Acqknowledge* software.

### Component 3: feedback model

The feedback model serves to select the optimal adjustment of the stressor game characteristics, i.e., the adjustment that minimizes the difference between the current stress state and the desired stress state. To this end, it models the relations between the size of the stress response types selected in the previous section and the stressor game characteristics adjustments chosen in the preceding section. These relations are expressed in rules that are fitted for each individual subject during the fitting condition of the application. It is important to realize here that the formulas presented in this section can be used to calculate values to a precision that does not necessarily reflect the same precision of the entities these are presenting, i.e., the provided formulas and resulting values in this section should be seen as an approximation of the respective entities these present.

#### Stress state

In correspondence with our definition of the stress state as the ensemble of responses to internal or external stimuli that present (perceived) challenges to the (psychological) homeostasis, the stress state in the feedback model is expressed as the weighted summation of stress responses in the selected stress response types (i.e., heart rate, heart rate variability, blood pressure, and electrodermal response). In order to estimate this stress state, we calculate the *physiological activity state.* This latter state is derived by calculating the activity for each stress response type separately, expressed in the standardized mean difference effect between the baseline and stress response values, denoted as Hedges' *g* (Hedges, 1981). The formula for the size of the activity of a given stress response type expressed in *g* is given by:

$$g=\frac{\mu_{\text{stress response}}-\mu_{\text{baseline}}}{\sigma_{\text{baseline}}}$$

Here μ_baseline_ refers to the mean value of the specific stress response type (e.g., heart rate) during a baseline measure. This baseline measure is a measure of the physiological activity state of the subject in rest, before the start of the respective paradigm. This measure needs to be performed shortly before the application is used, while the subject is instructed to relax in the same position as he or she will be in when using the application. Furthermore, the μ_stress response_ and σ_baseline_ refer to the mean current physiological activity of a given type and the standard deviation of the corresponding baseline measurement values. In this manner, the sign of the resulting *g* will be positive when the physiological activity is higher compared to the corresponding baseline measurement, with the value indicating the change from baseline expressed in standard deviations. The physiological activity state, calculated as the weighted summation of these stress response types is given by:

$$p_{c}=\sum \left(g_{i}*w_{i}\right)\;/\;\sum w_{i}$$

Here *p*_*c*_ stands for the current physiological activity state, summing over all *g*_*i*_, the current size of the respective stress response types, multiplied by *w*_*i*_, representing the respective weights assigned to these types. These weights are introduced to allow specific stress response types to have more influence on the physiological activity state than other response types, by assigning the respective weight of this response type a higher value than other weights. This can be desirable in certain therapy or research paradigms in which specific stress response types are more informative for the aim of the paradigm than others. By default the weights are all initialized to the value of 1, resulting in all stress response types having the same influence on the physiological activity state. The state is normalized by dividing it by the summation of all weights. In this manner the normalized weights sum up to one, resulting in a physiological activity state *p*_*c*_ that is expressed in the weighted average standard deviations change from baseline, making interpretation more intuitive. Calculating *p*_*c*_ in this manner allows to combine multiple stress response types, takes personal variance in physiological signals of individual subjects into account and makes sure that the physiological activity state value is close to 0 at the beginning of each experiment, by using the baseline measurements.

We use this physiological activity state to estimate the current stress state *s*_*c*_. Based on the current stress state estimated in this manner and the given desired stress state *s*_*d*_, the feedback model selects a stressor characteristic adjustment that is predicted to result in a stress state that is closest to the desired stress state. In order to determine this adaptation, the feedback model utilizes *rules* that model the relation between stressor characteristic instantiations and the different stress response types.

#### Rules

For every stressor characteristic instantiation, the feedback model contains exactly one rule that models the relation between all the available adjustments of the instantiation and the corresponding responses in the different stress response types. For example, for the stressor characteristic “game music” the feedback model contains a rule that predicts the response for each of the different stress response types (i.e., heart rate (variability), blood pressure and the electrodermal response) for each of the possible adjustments, i.e., for each music sample that can be presented.

Two kinds of rules are contained in the feedback model: *discrete* and *continuous* rules. The discrete rule is used for modeling relations concerning stressor game characteristics that are instantiated in discrete levels, such as realism, which is instantiated by adjusting the point of view to either the first or the third person view, i.e., two levels. This kind of rule is represented as a matrix ***D***_*ij*_, with the rows representing the change in the size of the respective response types, and the columns representing the transitions between levels of the stressor game characteristic instantiation. There are *i* rows, equal to the amount of stress response types, and *j* columns, equal to the number of 2-permutations of the set of levels of the stressor game characteristic [i.e., P(*amount of levels*, 2)], representing all the possible transitions from one level of a given characteristic to another. In this manner the element *D*_*ij*_ refers to the predicted change in response type *i*, i.e., Δ *g*_*i*_, when the stressor game characteristic transition is applied that belongs to column *j.* For example, the rule for transitions of the realism characteristic instantiation would consist of a four by two matrix, with the four rows presenting the changes in size of the different stress response types and the two columns representing the two possible transitions: from first to third point of view, and vice versa. Within this presentation, the predicted change in the current stress state, i.e., $\hat{△s_{c}}$, when applying transition *j* on the stressor game characteristic presented by discrete rule *r*_*d*_, is therefore equal to the weighted summation (using the weights corresponding to the respective stress response types) of the elements in column *j* in matrix ***D***:

$$r_{d}(j)=\hat{△s_{c}}\;with\;\hat{△s_{c}}=\;\sum_{i}\left(D_{ij}*w_{i}\right)$$

The continuous rule is used for modeling relations of stressor characteristics that are instantiated in a continuous manner, such as aversive stimuli, which is instantiated by using sounds and pictures from the IAPS and IADS using a continuous scale. This kind of rule consists of a simple linear regression model for each response type, in which the respective predicted response sizes are regressed on the continuous measure of the stressor characteristic. This entails that the predicted change in the current stress state, $\hat{△s_{c}}$, when applying an adjustment with value *x* of the continuous measure of the stressor game characteristic instantiation presented by the rule *r*_*c*_, is equal to the weighted summation of the predicted changes in stress response type sizes, Δg_i_. These changes are equal to the value predicted by the fitted regression model:

$$\begin{array}{l} r_{c}(x)=\hat{△s_{c}}\;with\;\hat{△s_{c}}=\sum \left(\hat{△g_{i}}*w_{i}/\sum w_{i}\right)\\ \;with\;\hat{△g_{i}}=\beta_{0}+\;\beta_{1}\cdot x \end{array}$$

In the example of the instantiation of aversive stimuli in the form of pictures from the IAPS, the desired change in the current stress state can be inserted, resulting in a value for *x* that corresponds to the value of the scale used for aversive pictures that is predicted to result in this desired change of stress state. Subsequently, the picture with the value closest to *x* is selected to be used as stressor game characteristic adjustment.

In order to select the stressor characteristic adjustment that is predicted to result in the stress state that is closest to the desired stress state, the feedback model will inspect the predicted stress state change of all rules when applied on the current stress state. Subsequently it will select the rule that results in the stress state that is closest to the desired stress state *s*_*d*_ and apply this rule, i.e., stressor characteristic adjustment, to the stressor game. This entails that one rule is executed at a time. When we consider all possible rules *r* with all possible input values *x* or *j* as a set *R*, this selection process is given by:

$$\underset{r(x\vee j)\;\in \;R}{\text{min}}\{\;s_{d}-(s_{c}+r(x\vee j))\}$$

In cases where two or more rules would result in identical predicted stress states that are both the closest to the desired stress state, the feedback model selects the rule that has the largest effect on the stress response type that has the value the farthest away from the desired stress state. This is applied to prevent flooring and ceiling effects of stress response types.

#### Fitting

In order to derive the rules of the feedback model for each individual subject, the subject is presented with a predetermined sequence of stressor characteristic adjustments in the fitting condition, i.e., the condition where the subject searches for notes in the maze in the garden. This sequence can be specified by the therapist or researcher, allowing to determine which rules will be used and therefore included in the feedback model and how many times and which specific adjustment will be presented.

The response to the adjustments are calculated by using the measurements x seconds before the adjustment as a baseline and the measurements y seconds after the adjustment as response, after which *g* is calculated for each stress response type as described in section Stress state. The values of x and y can be chosen by the therapist or researcher in order to allow for specific intervals of measurement suited for the specific stress response types being used. Because we only adjust one stressor characteristic at a time and keep all other variables that we identified as being relevant to the response constant, and do so in a homogeneous environment (the maze looks virtually the same at any given time), we take the measured response as representing the expected change in the stress response types after applying this specific adjustment. Although we aim to control all relevant variables and solely adjust the characteristic of interest, we would recommend therapists or researcher creating a sequence of adjustments to use multiple presentations of a specific adjustment, resulting in multiple-trial measurements in the fitting condition. In this manner, any remaining effect of variation in non-controlled variables on the measured response will be reduced.

The measured sizes of the response types to these stressor characteristics are used to fit the feedback model rules. For the discrete rules, this entails creating a matrix of the possible transitions between the levels together with the measured responses. The continuous rules are derived by constructing a simple regression model using least squares regression estimation (LSRE), with the measured responses as data to fit the regression model.

#### Intervening variables

Intervening variables, i.e., variables influencing the relation between the stressor and stress response, can intervene with the stress state that is predicted by a rule executed by the feedback model. In these cases, the feedback model cannot reliably predict, and therefore not control, the stress state. Currently, the feedback model controls for two kinds of intervening variables.

First, the model controls for intervening variables of the individual difference variables category, such as gender and age, by fitting an individual feedback model for every subject separately.

Second, the feedback model aims to reduce intervening flooring and ceiling effects of stress response types by selecting rules that have the largest effect on the stress response type that has the value the farthest away from the desired stress state.

#### Implementation

The feedback model is implemented in Python (van Rossum, 1995), as this language provides classes for interaction with the measurement component software *Acqknowledge* and is a high-level programming language, allowing easier adaptation in the future when this is needed for a given paradigm.

### Overview

**Figure 3:**
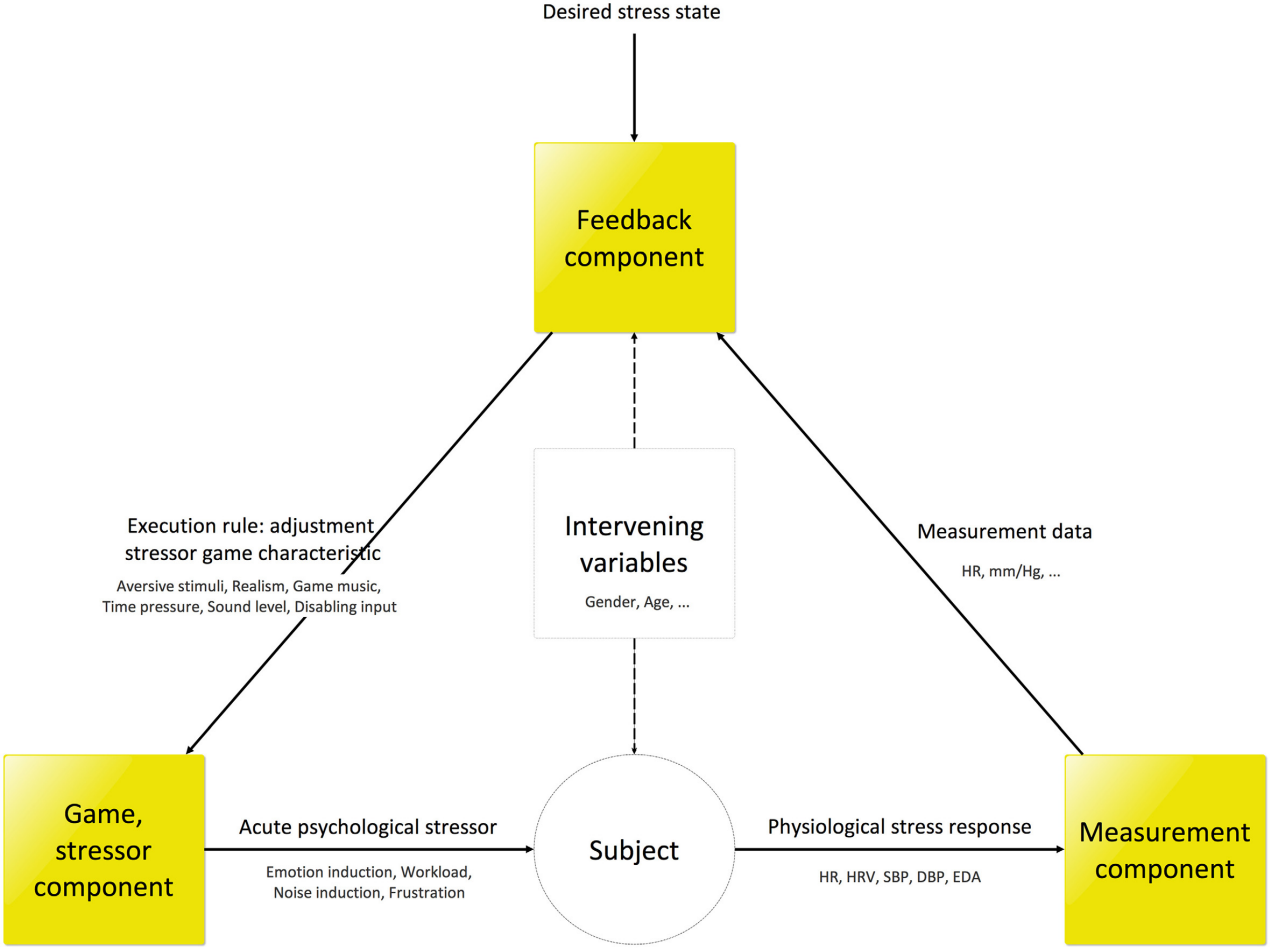
**Overview of application**.

In Figure 3 the schematic overview GASICA from Figure 1 is revisited, and elaborated with the outcomes of the top-down design process of the different components as described in the previous sections. In this figure the constant loop performed in GASICA is depicted. In this loop the digital stressor game presents various stressor forms such as emotion induction, workload and frustration to the subject (Table 1) through the adjustment of (instantiations of) stressor game characteristics, e.g., the adjustment of time pressure through the presentation of a countdown. Simultaneously, five different physiological stress response types (i.e., heart rate, heart rate variability, diastolic, and systolic blood pressure, and electrodermal response) of different forms (respectively cardiac, hemodynamic, and sympathetic) are measured (Table 3). These measurements are relayed to the feedback model, which determines the current stress state and selects a rule (resulting in a adjustment of an instantiation of a game characteristic) that will minimize the difference between the desired stress state and the predicted current stress state after applying the rule. This rule is then applied in the stressor component, resulting in an adjustment of the game characteristic instantiations, hereby closing the loop that will run continuously. Because this loop runs continuously, any changes in the current stress state that are not a consequence of the adjustment of stressor characteristics (e.g., spontaneous drifts or influences of non-controlled variables) are constantly measured and corrected for. A narrative is used in the stressor game to prevent subjects to identify adjustments as part of the therapy or research intervention: the subject has to find notes wearing a heavy suit that contains a radio and display, and which malfunctions from time to time, resulting in for example, the presentation of images, sounds, and the restriction of movement. Intervening variables are taken into account by the feedback model in two ways, most prominently by constructing an individual feedback model for each subject.

Important to realize here is that GASICA utilizes single stressor characteristic adjustments to alter the stress state. However, the envisioned workings of GASICA do not rely on the expectation that single adjustments will result in the exact responses as found during the fitting condition. Because we *continuously* alter different characteristics, we get an ensemble of stressor manipulations that together have more power to alter the stress state toward the desired stress state. In other words, it is not the case that each specific, relatively mild, stressor is expected to elicit the exact response measured during the fitting, but rather the ensemble of all stressor characteristics that are continously presented based on the current stress state, that is expected to result in the alteration of the stress state toward the desired stress state.

Instructions provided to subjects can be altered to fit the needs of the respective paradigm being used. Two general instructions that need to be given in any paradigm are that (1) the subject should perform to the best of their abilities, i.e., find as many notes as possible (monetary incentive could be employed here), and (2) the subject should remain as still as possible in order to ensure valid physiological meaurements. Important to note here is that due to the first instruction, GASICA is expected to also present a certain amount of social-evaluative threat, as the subject feels that they are evaluated on how well they are performing.

Most of the properties of GASICA can be altered by the therapist or researcher using it, as indicated in relevant sections in this manuscript. In this manner we aim to present a generic application that can be used in a multitude of paradigms. Some of the most important alterations include:

- Which elements of a paradigm, such as a baseline measurement, a fitting condition and a manipulation condition are presented, and in which order.
- Determining the used rules and corresponding stressor characteristics.
- Which characteristics are presented during the fitting condition, in which order, and how many times.
- Whether the manipulation condition utilizes a predetermined sequence of stressor characteristic adjustments or utilizes the feedback model fitted during the fitting condition.
- If a task or therapy element will be presented during the manipulation condition and how this is alternated with the characteristic adjustments.
- How long the baseline measurement will be.
- Which stress response types are measured and used to determine the stress state, and the corresponding weights.

#### Implementation

**Figure 4:**
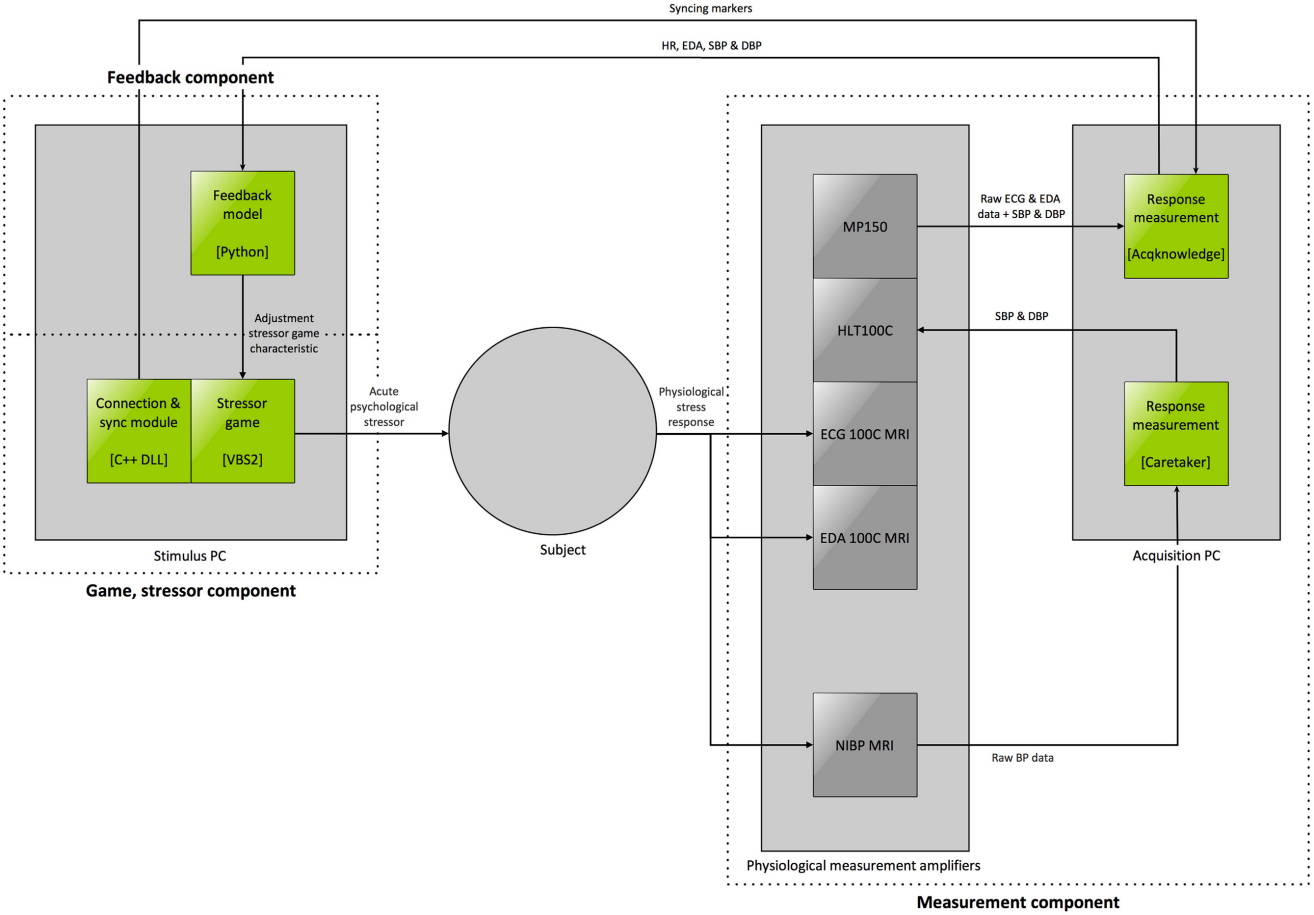
**GASICA architecture**. Gray elements represent hardware such as computers and amplifiers, green elements represent software, with the software environment or language indicated between brackets. The main components from Figure 3 are indicated with dotted lines.

In Figure 4 the complete architecture of GASICA is given, containing the implementations of the different components and the implemented connections between the components. The stressor game environment (VBS2) and the feedback model (Python) are run together on one pc, the *stimulus pc*, and the stress response measurement software (Caretaker and Acqknowledge) is run on another pc, the *acquisition pc*. This distinction is made because the software on the different pc's has different requirements: the stressor game environment requires more graphical power, whereas the measurement software mostly requires large memory and fast writing to the hard disk. By separating the components, we can utilize pc hardware that is better suited for different software, and prevent interference between the software packages. Furthermore, an additional module, the *Connection and sync module*, is developed as a dynamic link library (DLL) in C++ and is utilized as a plugin in VBS2, allowing to connect additional measurement equipment to GASICA using either the parallel or the serial port. Additionally, this module serves to synchronize the measurements in the measurement component with events in the stressor game or in any additional tasks that are used in different paradigms.

**Figure 5:**
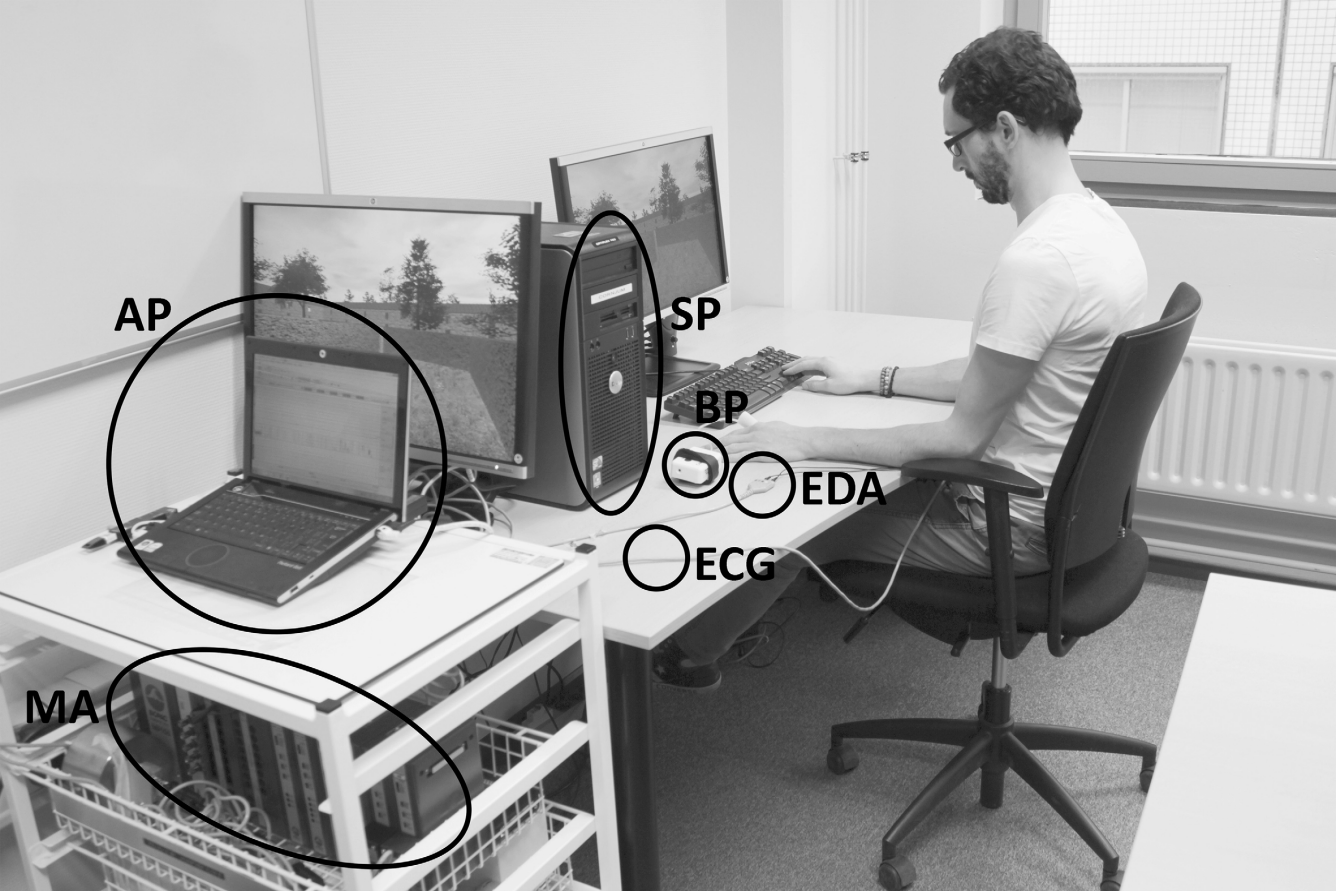
**Picture of GASICA in use, with the different components encircled**. AP stands for acquisition pc, SP for stimulus pc, BP for blood pressure measurement, EDA for electrodermal activity measurement, ECG for electrocardiography measurement and MA for measurement amplifiers.

Furthermore, the therapist or researcher can determine several properties of GASICA as described above. In order to facilitate this adjustment, all properties can be set through a single configuration file. Even more, all software components are coded as modular open-source code and will made available in due time on gasica.com. This allows therapist or researchers to change any element of the application that could not be altered using the configuration file, for example the way the stress state is determined, or the game design. In Figure 5 a picture of GASICA in use is included, with the different components from Figure 4 encircled.

## Discussion

We have presented GASICA, an application aimed at controlling the internal stress state in various therapy and research paradigms by online and continuous monitoring of the stress state through (neuro)physiological signals. Here we discuss the strengths and limitations of the application, and the future directions.

### Strengths

Through the fulfillment of the requirements of the different components, GASICA is an application that presents several strengths:

1. Multidimensional
  The application allows to present different stressor forms, such as workload, emotion induction, and frustration. This allows to investigate the effects of these specific stressor forms, in isolation or in combination, in different paradigms.
2. Ecological valid
  The stressor forms presented and the instantiations of the stressor game characteristics that present these forms allow for the presentation of ecological valid stressors. This allows for the execution of paradigms that produce results with more generalizable power to real-world situations.
3. Controllable
  The application allows to both control the stressor form and stressor intensity that is presented, aiming to result in the control of the stress state of the subject. If this succeeds, it opens many possibilities. For example, it allows to use GASICA to keep the stress state of the subject within desirable bounds given a specific paradigm, for example in therapies such as exposure therapy. Another example is to use the application to keep the stress state of a subject on a certain level for the duration of the paradigm, relevant, for example, in cases where the stress state is an intervening variable on the dependent variable in research.
4. Generic
  The application is generic in several respects. First, the configuration file allows to adjust any of the properties of GASICA, such as the adding of tasks to the application (the task itself can be constructed using the high-level programming language of VBS2), or the possibility to exclude or include stressor game characteristics and alter existing ones, allowing to present the stressor forms that are required in the respective paradigm. Second, the narrative and 3d world can be adjusted (the environment ensures easy import of existing 3d worlds and objects). Third, a wide range of physiological measurements can be used by adding Biopac amplifiers through a simple plug-and-play interface. After the new amplifier is added, the complete GASICA application automatically detects this new signal and utilizes it for control of the stress state. Additionally, measurement equipment from other vendors can be added through the connection and sync module. These properties allow adjustment of the application to make it suitable for different therapy and research paradigms. Furthermore, the entire application will be provided as modular open-source software on gasica.com, in order to allow any adjustments that are not feasible through the configuration file. This generic nature also allows GASICA to be used in additional ways. Examples could be to add neural activity measurement devices and make the measurements from these devices available to the subject, hereby effectively using GASICA as a neurofeedback application. Other examples could be in the entertainment field, where GASICA can be used to optimize the user experience, or for training purposes, aimed at the training of functioning in stressful jobs, such as in the military, aviation, or firefighting.

### Limitations

Several limitations can be anticipated with this application. First, it is to be expected that the rules fitted during the fitting condition capture a relation between stressor and response that is context-dependent, for example dependent of the elapsed time the stressor has been presented. As such, these relations are prone to change during the course of the experiment or therapy. At this moment, the application does not control for this. We aim to investigate these effects during the upcoming validation study and determine possible solutions, such as using *adaptive rules* in the feedback model, i.e., rules that adjust to changing relations between stressors and responses.

Second, currently only a few intervening variables are controlled for in the application. In the upcoming validation study we will analyse the effect of several intervening variables, such as elapsed time of the current paradigm, and include these in the feedback model.

Third, the application utilizes linear regression models to model the relation between continuously instantiated stressor game characteristics and stress response type sizes. However, it is not certain whether this relation is linear. We will use the data from future research to assess what kind of model best fits these relations.

### Stress induction

One of the important questions regarding the use of GASICA is whether it induces stress, or that the found responses are due to other concepts, most prominently, arousal. As stated in the treatment of these concepts in the introduction, we concur with the distinction proposed by Day and Walker (2007). We feel that according to this distinction GASICA should be considered as a stressor, as GASICA presents many stimuli that have been found to be aversive stimuli, entailing both qualitative appraisal in terms of aversiveness, and meeting the requirement that aversive challenges must be utilized.

Given that it is pivotal to establish with the highest possible certainty that the responses to GASICA are indeed representing stress, we have planned a follow-up validation study to this Technology Report. In this study a large study population is used, containing a control group to control for other factors of the digital stressor game that can contribute to physiological responses, such as motor activity. Furthermore, additional measurements are included, such as additional subjective measurements and cortisol measurements. As cortisol is an important and widely used biomarker for stress that could not be used as an online measurement in the measurement component, we will use it as an offline measurement in this study to gain more insight in the effects of GASICA on stress.

### Conflict of interest statement

The authors declare that the research was conducted in the absence of any commercial or financial relationships that could be construed as a potential conflict of interest.

## Footnotes

^1^The search phrases used were *game AND physiolog*^*^ and *stress^*^ AND game*, where *AND* indicates a logical conjunction and the asterisk (“^*^”) indicates a wildcard, i.e., any combination of characters. The search was performed in Pubmed, Scopus, PsycInfo, and the IEEE Xplore Digital Library.

^2^Heart rate and heart rate variability are measured using electrocardiography (ECG); systolic, diastolic and mean blood pressure are measured using a blood pressure monitor; electrodermal response is measured using electrodes on the skin; cardiac output, pre ejection period, cardiac index, left ventricular ejection time and vascular rigidity index are measured using impedance cardiography (ICG); digital blood volume pulse is measured using a photophlesmograph; and neural spectral power or event-related potentials are measured using electroencephalography (EEG).

^3^These mean effect sizes represent the difference between baseline measurements and measurements during digital stressor presentation.
